# Dermatologic Microsutures Using Human Hair: A Useful Technique in Cutaneous Stitching

**Published:** 2017-08-21

**Authors:** Mohammed Al azrak, Rei Ogawa

**Affiliations:** ^a^Burn & Plastic Surgery Unit, Surgery Department, Fayoum General Hospital, Fayoum, Egypt; ^b^Department of Plastic, Reconstructive & Aesthetic Surgery, Nippon Medical School, Tokyo, Japan

**Keywords:** dermatologic sutures, cutaneous wounds, stitching technique, hair stitching, hair microsutures

## Abstract

**Background:** Facial wounds are challenging for dermatologic surgeons, particularly traumatic facial wounds, because they can yield disfiguring scars. To obtain good results, narrow needles and sutures are needed. Hair filaments have a very small diameter (0.06-0.1 mm) and could serve as suture threads for facial wounds. **Objective:** To determine the aesthetic outcomes by using autologous hair to suture facial wounds. **Patients and Methods:** This case series study examined the aesthetic outcomes of all consecutive female patients with traumatic facial wounds who underwent autologous hair-based stitching in 2009-2016. Autologous hair ampoules were generated from an insulin needle. Micro instruments were used for wound stitching. **Results:** In total, 54 females (mean age, 10.8; range, 3-45) years had 56 traumatic wounds. Mean wound length was 3.6 (range, 1-12) cm. Injury depth varied from cutaneous-only to muscle involvement. Suturing yielded good edge coaptation, nice healing, and excellent aesthetic outcomes; the scars were often scarcely visible. Suture marks were not detected. Cutaneous reactions did not occur. **Conclusion:** Autologous hair can serve as a thread for closing facial wounds. It is low cost and thus suitable in settings characterized by facility and equipment limitations. It is also suitable for the battlefield.

The repair of traumatic facial cutaneous wounds carries a considerable risk of disfiguring scarring. Regardless of their age, females are significantly more affected by facial scarring than males because of social norms that value female attractiveness.^1^ Thus, when repairing traumatic facial wounds in females, it is necessary to make an effort to conserve their aesthetic appearance.

To obtain good aesthetic outcomes after wound repair, it is necessary to employ a variety of surgical techniques.^2^ Several local and technical factors have a considerable impact on the aesthetic outcome after repair of traumatic cutaneous wounds. One of the most important factors is the nature of the thread used for stitching, particularly its inability to provoke unwanted immune reactions by the body.^3^^,^^4^ In this case series, we used the scalp hair of the patients as a thread for stitching their traumatic facial wounds. In our experience, autologous hair did not elicit noticeable cutaneous reactions and yielded acceptable scars.

## PATIENTS AND METHODS

### Ethics statement

This work was conducted according to the ethical guidelines of the Declaration of Helsinki and its revisions and was approved by the institutional review board of Fayoum General Hospital. All patients in the case series provided informed consent to undergo the surgical procedures and to have photographs of their wounds/scars taken.

### Patients

This case series consisted of all consecutive female patients who presented with traumatic facial wounds at our tertiary care hospital (Fayoum General Hospital) in Egypt and underwent wound closure by stitching with autologous human hair between 2009 and 2016. While this technique was performed with male patients as well, we selected this subset of patients for case series review.

### Preoperative wound assessment

The wounds were assessed carefully during a physical examination, and the patient history was taken from the patient or guardian. During the physical examination, the length and depth of the injury were determined and the wound was assessed for injury to the nerves, blood vessels, ducts, muscles, and other structures. The presence or absence of hard tissue fractures was assessed by radiography. The patient or guardian was asked how the wounding occurred, with the aim of determining the risk of wound contamination. The patient or guardian was also asked whether the patient had any systemic conditions that could affect wound healing (eg, diabetes mellitus), whether previous wounds had left visible or abnormal scars, and whether the patient had a history of allergies or complications during anesthesia. The information that was retrieved during the physical assessment and discussion allowed the surgeon to determine the most suitable type of anesthesia and intervention. The patient or guardian was informed about the technique that would be used. The patient or guardian was also informed that it would be necessary to adhere to a rigorous follow-up regimen, namely, outpatient visits to the hospital every 3 days until the removal of the stitches, every 2 weeks in the first month thereafter, then every month for 2 months, and then every 3 months for at least 2 years. They were also told that photographs of their wound/scar would be taken at each visit. The patient or guardian was then asked to consent to the procedure and the taking of photographs. If the patient or guardian was literate, consent was obtained in writing. Alternatively, verbal consent was obtained.^5^

### Surgical technique

**Figure 1:**
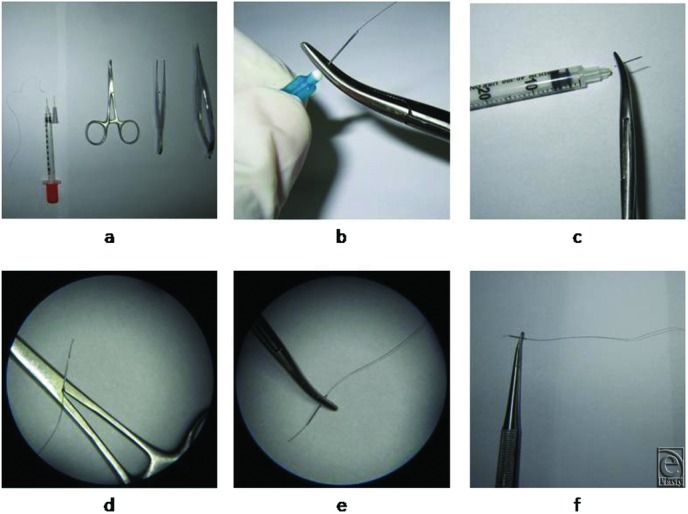
Tools needed for autologous hair-based suturing and the production of autologous hair ampoules. (a) The basic equipment needed for dermatologic microsuturing using hair filaments. (b-f) Production of an autologous hair ampoule. (b) The hair filament is passed through a 27-gauge insulin needle until it protrudes from the bevel end, after which the metallic part is separated from the plastic base by holding the metallic part with a mosquito or artery forceps and bending it from side to side until it breaks off the base. (c) If a 30-gauge insulin syringe is used instead of a 27-gauge insulin needle, the metallic needle can be separated from the syringe with a similar sawing method. (d) To anchor the hair filament to the metallic part of the needle, 1 mm of the needle base is crushed by using small mosquito forceps. (e) The view under loupe magnification (5.5×) of a 30-gauge needle with a hair filament anchored to it and grasped by a micro needle holder. (f) The hair filament is stretched straight, and the needle is grasped by the micro needle. The construct is now ready for cutaneous stitching.

The surgery involves the use of sterile latex gloves, narrow needles (27 or 30 gauge), mosquito or artery forceps, a micro needle holder, and small forceps (Fig 1*a*). All patients underwent stitching in an operating room to ensure the sterility of the procedure.^6^ Depending on the nature of their wound, the patients were given the most appropriate anesthesia, namely, local injection alone^7^ or combined with intravenous sedation or general anesthesia.

### Production of an ampoule of hair thread

To generate the ampoule of hair thread, select long durable hair filaments from the scalp of the patient and separate them by scissors. Fed the filaments into the insulin needle (27 or 30 gauge) in a retrograde direction. Before or after that, separate the metallic needle from the attached plastic part (Figs 1*b* and 1*c*). Leave a small part of the filament inside the needle. Crush 1 to 2 mm of the base of the needle by placing it between the 2 arms of the hinge of a mosquito, and compress the handles until the ratchets lock (Fig 1*d*). Now the hair filament anchored to the needle forming an ampoule for suturing is obtained (Figs 1*e* and 1*f*).

Video.

### Sterilization of the ampoule of hair thread

The hair filament anchored to the insulin needle can be sterilized by immersing the ampoule in a small gallipot or container filled with alcohol alone or a mixture of alcohol and betadine for 10 to 30 minutes.^8^ It should be noted that the ampoule should be prepared in an infection-controlled environment to prevent infection.^9^

### Wound preparation

After administering the anesthesia, the wound was washed by saline and antiseptic solutions such as povidone-iodine or chlorhexidine.^10^^,^^11^ Foreign bodies were removed when they were found. The integrity of the adjacent vital structures was checked, and essential repair was performed as required.^12^ Unviable tissues were debrided meticulously and conservatively until healthy tissue was achieved.^13^ Hemostasis was ensured before starting wound closure.

### Stitching technique

Essential deep suturing techniques were applied in the wounds in some cases when needed by using 3-0/4-0 polydioxanon sutures (PDSII) and/or polyglactin sutures (Vicryl).^14^^,^^15^ The wound was then closed with simple interrupted or continuous autologous hair sutures^16^^,^^17^ that precisely approximated the wound edges. Thus, under surgical loupe magnification, the epidermis and the dermis were first gently penetrated with the straight insulin needle (30- or 27-gauge). Traction was applied on the thread to help pass all of the remaining part of the filament through the epidermis except for a few centimeters, which would be used later to generate the surgical knot. The opposite edge of the wound was then penetrated, placing traction on the thread and not the needle. This is because dragging the thread through the epidermis by continuous traction on the needle may cause the hair filament to detach from or break off at the needle base. The surgical knot was then easily generated by using a fine needle holder aided by fine forceps (toothed or untoothed). The proper alignment of the epidermal layer was checked under loupe magnification.

### Wound care after surgery

Topical antibiotics were applied on the sutured wound to prevent infection, and the wound was covered with a sterile dressing.^18^ The patient was discharged from hospital and asked to return 3 days later. At the first visit, the dressing was removed so that the wound would be left exposed. The patient was asked to apply topical antibiotics daily and return to the hospital 3 days later. At that second visit, the wound was checked. If the wound had not yet healed reliably, the patient was asked to return to the hospital in another 3 days. This procedure continued until reliable healing was achieved. At that point, the stitches were removed.^19^^,^^20^

The patients were instructed to return to the hospital every 2 weeks in the first month and then every month for 2 months.^21^ Thereafter, the patients were asked to return to the hospital every 3 months for at least 2 years.

The stitched sites were evaluated at each visit by keen inspection with the naked eye, examination under a dermatologic lens, and examination under a surgical loupe at 5.5 × magnification. The photographs that were taken at each visit were also examined by using the photoviewer program at various magnifications (up to 10×).

## RESULTS

### Patient and wound characteristics

**Figure 2:**
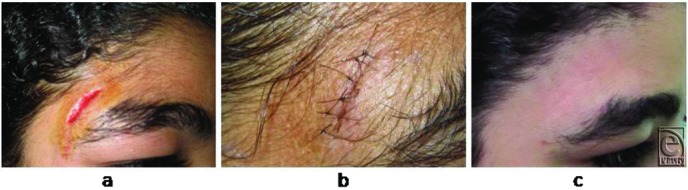
The facial wound before and after stitching and the scar at 6 months in case 1. (a) This 14-year-old girl had a simple facial cut wound that was 3-cm long. (b) The view 1 week after stitching with an autologous hair filament. (c) Six months later, there is no marked disfiguring scar. Suture marks cannot be seen.

**Figure 3:**
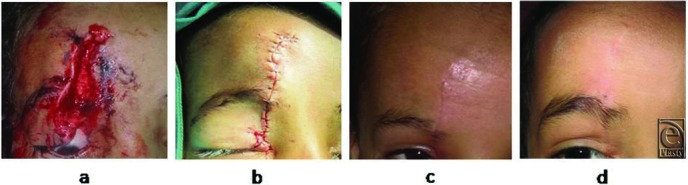
The facial wound before and after stitching and the scar at 5 weeks and 6 months in case 2. (a) This 7-year-old girl had a deep facial wound that was 6-cm long and involved injury to the skin, subcutaneous area, and muscles. (b) The view immediately after deep wound repair and epidermal stitching with an autologous hair filament. (c) The view 5 weeks after stitching. (d) The view 6 months after stitching. There is good healing and no marked disfiguring scar or suture marks.

**Figure 4:**
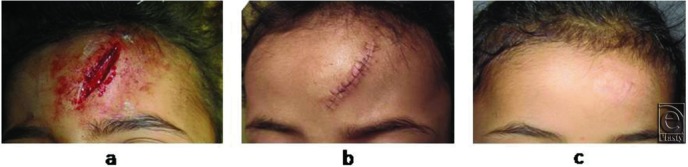
The facial wound before and after stitching and the scar at 3 months in case 3. (a) This 6-year-old girl had a deep facial wound that was 4-cm long. (b) The view 6 days after suturing with superficial autologous hair sutures. (c) The view 3 months after stitching. A nice healing line can be seen. There are no suture marks.

The case series consisted of 54 females with 56 facial traumatic wounds. All patients were Egyptian (Afro-Mediterranean race) and their average age was 10.8 (range, 3-45) years. The average length of the wounds was 3.6 (range, 1-12) cm. The depth of the wounds ranged from being only cutaneous (9 wounds, 16.1%) to involving both the skin and subcutaneous tissues (40 wounds, 71.4%) to reaching the muscles (7 wounds, 12.5%). The wounds were either pure cut wounds caused by sharp objects (including knives used in acts of violence or the sharp edges of broken glass that were encountered accidentally) or lacerations or contusions caused by the blunt edges of crushing objects (see panel *a* in Figs 2-4). The most common locations of the wounds were the lateral forehead (*n* = 14; 25.0%), followed by the periorbital region (*n* = 13; 23.2%), the mid part of the forehead (*n* = 8; 14.3%), and the perioral region (*n* = 7; 12.5%) (Table 1).

### Surgical characteristics

For anesthesia, most patients (*n* = 41; 75.9%) underwent local injection combined with intravenous sedation. The remainder underwent local injection alone (*n* = 7; 13.0%) or general anesthesia (*n* = 6; 11.1%).

In terms of stitching, most wounds (*n* = 47; 83.9%) received simple interrupted stitches and surgical knots. The remaining 9 wounds (16.7%) received simple continuous sutures.

### Follow-up

The sutured wounds were examined every 3 days on average, and the stitches were removed 6 to 15 days after closure. A few patients did not follow the follow-up timetable properly. As a result, the stitches were sometimes left in place for 2 weeks, without any apparent ill effects.

### Wound outcomes

In all cases, there was good coaptation of the wound edges along the length of the wound and the scars that were left were fine lines that matched well with the surrounding skin in terms of color and texture (Figs 2-4). Dehiscence and infection were not detected in any of the wounds/scars. Suture marks across the healing line were not detected in any of the cases even after 2 years of follow-up. The patients and their relatives were generally satisfied with the aesthetic outcomes of the stitching.

### Observations during suturing with hair

In all cases, the hair was pliable and smooth, provided effective tension, and harmonized with the skin tissue. The knot was secure and did not exhibit dehiscence. The stitches accommodated the local wound edema and did not cut through the edematous tissue. The stitches were easy to remove. The patients appeared to feel less pain on suture removal than our experience with other suture materials. Cost and availability issues were not encountered.

## DISCUSSION

Scarring is a crucial hallmark of skin reconstitution. Since only humans scar, there are few animal models of scarring as well. This has significantly hampered our understanding of the mechanisms that underlie wound healing and normal and abnormal scar formation. Nevertheless, recent research has improved our understanding of these mechanisms.^22^^-^24 In particular, we are now aware that the size, texture, and color of scars are influenced by various cells and local growth factors.^25^ However, the clinical approaches that best prevent or ameliorate scarring remain unclear. As a result, patient satisfaction with the aesthetic results of wound closure still varies markedly.^24^ In particular, given the social consequences of facial disfigurement, the aesthetic outcomes of stitched wounds on the face can give rise to patient dissatisfaction. Thus, it is essential to handle facial wounds meticulously.

Our case series analysis showed that using autologous hair for suturing facial wounds resulted in excellent aesthetic outcomes: the wounds were often invisible or scarcely visible after only a few months of follow-up, and most of the patients and their relatives were satisfied with the outcome. It should be noted, however, that to prevent scar prominence, patients should be encouraged to protect themselves from sun exposure after hair-based suturing.

We found that the hair-based sutures did not generate any unwanted cutaneous reactions. This may reflect the fact that the hair was derived from patients themselves; thus, it was not recognized as a foreign body and did not trigger wound inflammation, which can yield unappealing large and reddened scars. However, further basic studies on the immunogenicity of autologous hair-based sutures are warranted.

During facial suturing, we paid particular attention to possible nerve injuries and ensured that they were repaired. This reflects the research conducted by Yannas et al.,^26^^-^29 who studied the repair and regeneration of the skin and nerves and reported the similarities and differences between these 2 structures. Their observations showed that it is important to meticulously repair injured nerves at the cut site to prevent neuroma formation. Also, we were keen to meticulously handle the cutaneous cut site to decrease scar formation.

Our autologous hair-based wound closure approach is likely to be particularly useful in settings characterized by facility and equipment limitations, such as those experienced by second-world countries. It may also be suitable for the battlefield. Our technique involves 2 aspects. First, the surgery is atraumatic surgery because micro- or fine tools are used. In our case series, we used fine tools and instruments similar to the ones we usually use in microsurgery procedures. This significantly minimized tissue trauma, gave precise control during stitching, and facilitated the handling of the fine hair filament. Second, the hair filament is a low-cost natural monofilament that can be used instead of synthetic threads. Its fineness also obliges the surgeon to perform the surgery in a highly meticulous and delicate manner. In fact, on some occasions, it increased the skill of the surgeon in terms of fine handling of the facial skin wound.

We did observe variations in terms of the thickness and length of the hair filaments in our case series. For this reason, hair-based suturing was performed in 2 steps: the hair thread ampoule was generated first and then the wound was sutured.

It is also important that the wound should be cared for properly before and after stitching. Thus, the wound bed should be prepared properly by removing foreign bodies, performing conservative debridement, and ensuring hemostasis. After stitching, the wound should be managed with sterile dressings and topical antibiotics until the stitches can be removed.

We are experienced in using most (if not all) commercially available suturing threads.^30^^,^^31^ This experience together with our long-term case series results suggests that for superficial suturing, autologous hair filament can be an effective alternative to other materials. Thus, given its excellent mechanical properties as a suturing material along with its low cost and ready availability, this nonabsorbable and natural thread can be added to the panoply of suturing materials. It should be noted, however, that its effectiveness in terms of reducing poststitching scarring is likely to be maximized when micro- or fine instruments are used.

## CONCLUSION

This preliminary case series report shows that autologous hair can serve as a thread for repairing skin wounds. We described our meticulous approach to using this delicate tissue for epidermal suturing. The aesthetic outcomes of using autologous hair as a suturing thread for facial wounds were excellent, and most of the patients expressed satisfaction. Hair-based thread is also low cost, meaning that it is suitable in settings characterized by facility and equipment limitations due to economic circumstances. It may also be useful for the battlefield or exceptional periods as in mass casualty.

## Figures and Tables

**Table 1:** Location of the facial wounds of the patients

Location of the wound on the face	No. of wounds (%)
Forehead (mid part)	8 (14.3)
Forehead (lateral)	14 (25.0)
Periorbital region	13 (23.2)
Nasal (side wall, dorsum)	5 (8.9)
Cheek (inner part)	4 (7.1)
Cheek (outer part)	3 (5.4)
Perioral region	7 (12.5)
Lower jaw	2 (3.6)
